# Hypersensitivity Pneumonitis From Fire-Retardant Spray in a Patient With Multiple Lung Pathologies and Elevated Immunoglobulin E

**DOI:** 10.31486/toj.20.0163

**Published:** 2021

**Authors:** Rohan Madhu Prasad, Tyler Kemnic, Abdullah Al-Abcha, Akhil Sharma, Shilpa Kavuturu

**Affiliations:** Department of Internal Medicine, Michigan State University–Sparrow Hospital, Lansing, MI

**Keywords:** *Alveolitis–extrinsic allergic*, *chemical fire-retardant spray*, *cigarette smoking*, *immunoglobulin E*, *nonmetalworking fluids*

## Abstract

**Background:** Hypersensitivity pneumonitis, also known as extrinsic allergic alveolitis, is a pulmonary disease with large knowledge gaps, including etiology, pathogenesis, diagnosis, and treatment.

**Case Report:** A 58-year-old male with a pertinent history of recurrent *Mycobacterium malmoense* presented to a tertiary emergency department after 1 week of difficulty breathing. He also reported a productive cough and fevers. The patient was an active smoker and was recently exposed to chemical fire-retardant spray. Chest x-ray showed extensive bilateral pulmonary infiltrates. The tertiary center initiated cefpodoxime 200 mg twice daily for 5 days and home azithromycin for possible pneumonia. However, the patient returned the next day with worsening symptoms. After the patient transferred to our institution, physical examination revealed a hypoxic patient with bibasilar crackles and wheezes. Laboratory tests revealed elevated white blood cell count, sedimentation rate, and immunoglobulin E. Chest computed tomography demonstrated growth of a previously noted right upper lobe intracavitary lesion and new onset diffuse interstitial pulmonary ground-glass airspace opacities. Hypersensitivity pneumonitis panel demonstrated positive antibodies to *Aspergillus fumigatus* antibody precipitin 1 and *Micropolyspora faeni*. The patient was given oral prednisone and advice on proper respiratory precautions in the workplace.

**Conclusion:** This case illustrates that hypersensitivity pneumonitis can develop via chemical fire-retardant spray. Additionally, patients with a smoking history and elevated immunoglobulin E should be evaluated for severe forms of the disease.

## INTRODUCTION

Hypersensitivity pneumonitis (HP) develops from particle inhalation that causes an exaggerated immune response.^1^ However, many aspects of HP are unknown, including etiology, pathophysiology, diagnosis, and treatment. Additionally, the incidence and prevalence of HP are still undetermined, largely because of its various causative agents and because no uniform diagnostic criteria have been created. Farmer's lung disease, a common form of HP, has a reported annual incidence of 44 per 100,000 farmers.^2^ Moreover, 4% to 13% of interstitial lung diseases are thought to be HP.^2^ Typical symptoms of HP are fever, chills, dyspnea, dry cough, and fatigue. However, chronic HP may develop into interstitial lung fibrosis or slowly progressive chronic fibrosis.^3^ Smoking and elevated immunoglobulin E (IgE) have been linked with severe types of HP.^3,4^ However, the symptomatology and disease course between severe and nonsevere forms of HP have not been delineated.

## CASE REPORT

A 58-year-old male presented to a tertiary emergency department (ED) because of difficulty breathing. The patient's medical history included emphysema, *Mycobacterium malmoense* with chronic right upper lobe cavitation, chronic interstitial lung disease caused by asbestos, and resolved aspergillosis. For the recurrent *M malmoense*, the patient was compliant with an outpatient regimen of daily ethambutol 1,000 mg, moxifloxacin 400 mg, and azithromycin 250 mg. Social history was pertinent for being an active 20 pack-year smoker. Additionally, the patient worked at a factory that produces chemical fire-retardant spray, where, for the past 6 weeks, he admitted that he had not worn protective facial equipment, and he particularly recalled an incident when he directly inhaled the chemicals accidentally. He had also worked on a farm but only during his childhood.

At the tertiary ED, the patient stated that for the past 1 week he had experienced shortness of breath on exertion, cough with productive yellow sputum, fevers, fatigue, and mild weight loss. Chest x-ray showed extensive bilateral pulmonary infiltrates, primarily in the medial right upper lobe and mid lower bilateral lungs. The tertiary center diagnosed the patient with community-acquired pneumonia and discharged him with instructions to continue his home azithromycin and start cefpodoxime 200 mg twice a day for 5 days. However, he returned the next day with persistent, worsening symptoms and was transferred to our institution.

**Figure 1.**
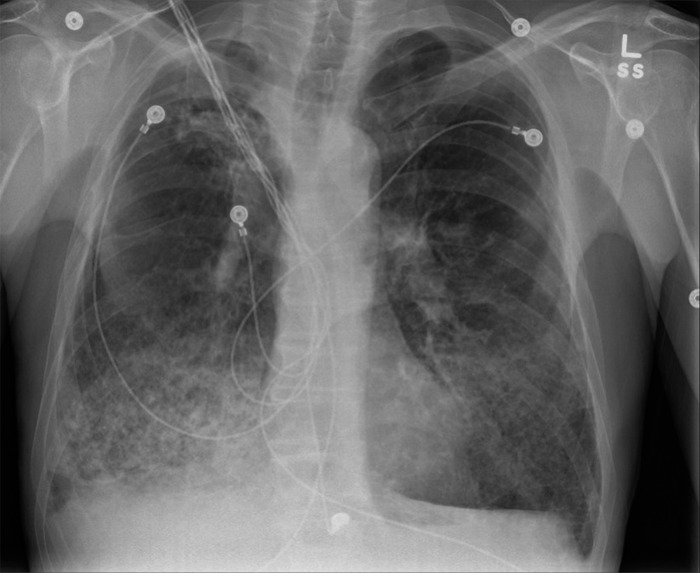
Admission chest x-ray shows bilateral infiltrates and chronic emphysematous changes.

**Figure 2.**
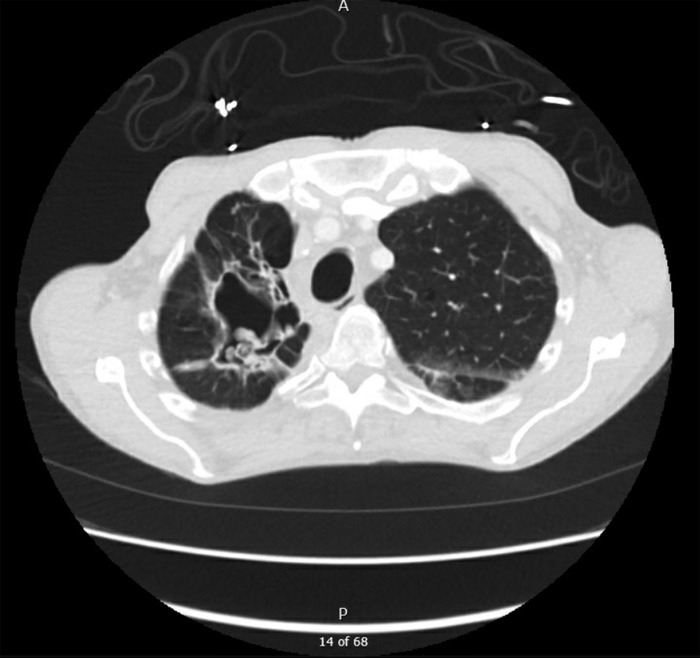
Admission chest computed tomography, transverse view of upper lobes, shows dominant right upper lobe intracavitary lesion. The central component is 2.1 × 2.3 cm (previously 2.0 × 2.3 cm). The mural nodule within this cavitation is 2.1 × 1.2 cm (previously 1.5 × 1.0 cm) and thin walled. Imaging shows progression of right upper lobe bullous emphysema and cystic bronchiectatic changes.

**Figure 3.**
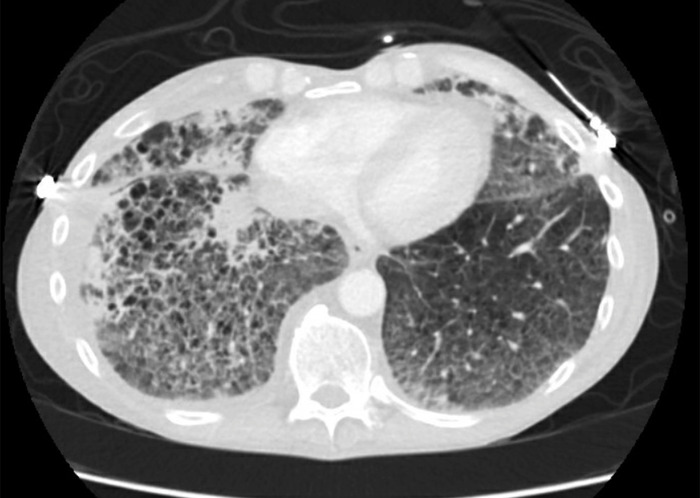
Admission chest computed tomography, transverse view of lower lobes, shows new onset diffuse interstitial pulmonary ground-glass airspace opacities.

**Figure 4.**
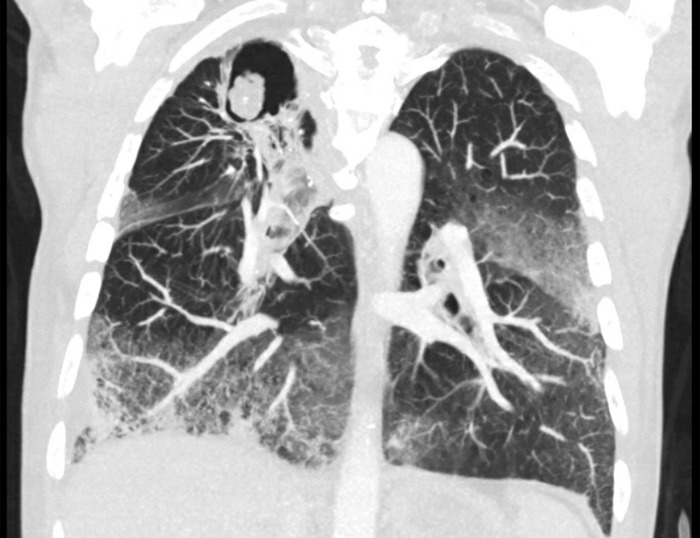
Admission chest computed tomography, coronal view, shows increased mediastinal lymphadenopathy, likely reactive.

In our ED, he was afebrile but hypoxic with an oxygen saturation of 82% on room air. Physical examination revealed bibasilar crackles and wheezes. Initial laboratory and microbiology investigations revealed elevated white blood cell count (29.2 × 10^3^ μL), sedimentation rate (57 mm/h), and total IgE (2,393 kU/L). Chest x-ray showed extensive bilateral pulmonary infiltrates (Figure 1). Chest computed tomography (CT) revealed growth of a previous right upper lobe intracavitary lesion (2.1 × 2.3 cm, previously 2.0 × 2.3 cm) and new onset diffuse interstitial pulmonary ground-glass airspace opacities (Figures 2, 3, and 4).

The patient was admitted to the hospital and started on supplemental oxygen and his home *M malmoense* antibiotic regimen. The pneumonia antibiotics were stopped. On day 2 of the hospital course, the patient received intravenous methylprednisolone 40 mg every 8 hours for 4 dosages. Thereafter, he was administered prednisone 40 mg daily until discharge. HP panel was positive for *Aspergillus fumigatus* antibody precipitin 1 and *Micropolyspora faeni* antibodies (Table 1). During the hospital course, no fevers were documented, the patient's shortness of breath improved, and his oxygen requirements gradually decreased.

**Table 1.** Admission Laboratory and Microbiology Investigations

Variable	Result	Institution Range
White blood cells, 10^3^/μL	29.2	4-12
Sedimentation rate, mm/h	57	0-15
Procalcitonin, ng/mL	0.06	0-0.09
Lactic acid, mmol/L	1.9	0.2-1.8
Total immunoglobulin E, kU/L	2,393	2.9-48.2
Hypersensitivity panel		N/A
*Aspergillus fumigatus* antibody precipitin 1 antibody	Positive	
*Micropolyspora faeni* antibody	Positive	
*Thermoactinomyces vulgaris* 1	Negative	
*Thermoactinomyces sacchari*	Negative	
*Aureobasidium pullulans*	Negative	
Pigeon serum antibody	Negative	
Acid-fast bacilli	Negative	N/A
Atypical pneumonia panel		N/A
*Chlamydia pneumonia*	Negative	
*Mycoplasma pneumonia*	Negative	
*Legionella pneumonia*	Negative	
*Streptococcus pneumonia* urine antigen	Negative	N/A
*Legionella* urine antigen	Negative	N/A
Methicillin-resistant *Staphylococcus aureus*	Negative	N/A
Influenza A/B direct antigen	Negative	N/A
Respiratory viral panel		N/A
Adenovirus	Negative	
Parainfluenza 1-4	Negative	
Metapneumovirus	Negative	
Respiratory syncytial virus	Negative	
Fungal precipitin panel		N/A
Blastomycosis	Negative	
Coccidioidomycosis	Negative	
Histoplasma H/M bands	Negative	
*Aspergillus* antigen	Negative	N/A
*Cryptococcus* antigen	Negative	N/A

N/A, not applicable.

Because of symptomatic relief, he declined high resolution CT, bronchoscopy, and biopsy for further diagnostic workup. The patient had multiple possible antigen sources that included his acute fire-retardant spray exposure, previous *M malmoense* infection, and remote farming history. However, during this admission, acid-fast bacilli smear and *Aspergillus* antigen were negative. The positive antibodies to *A fumigatus* antibody precipitin 1 and *M faeni* on the HP panel were considered incidental findings as the patient's symptoms did not develop until the acute exposure of the fire-retardant spray. The diagnosis of HP was further confirmed by leukocytosis, elevated IgE levels, bilateral pulmonary ground-glass airspace opacities on CT, and rapid resolution with contact withdrawal and steroids. Therefore, on day 3 of the hospital course, the patient was clinically diagnosed with HP because of chemical exposure from the fire-retardant spray.

**Figure 5.**
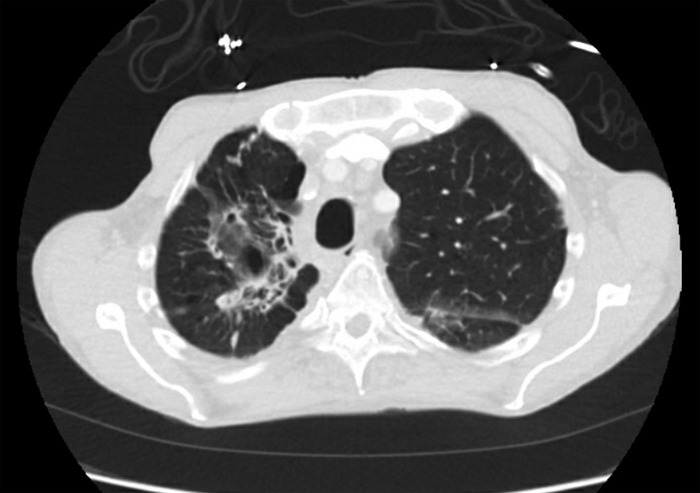
Follow-up chest computed tomography after 5 weeks, transverse view of upper lobes, shows the mural nodule in the right upper lobe intracavitary lesion at 3.1 cm and partially calcified.

**Figure 6.**
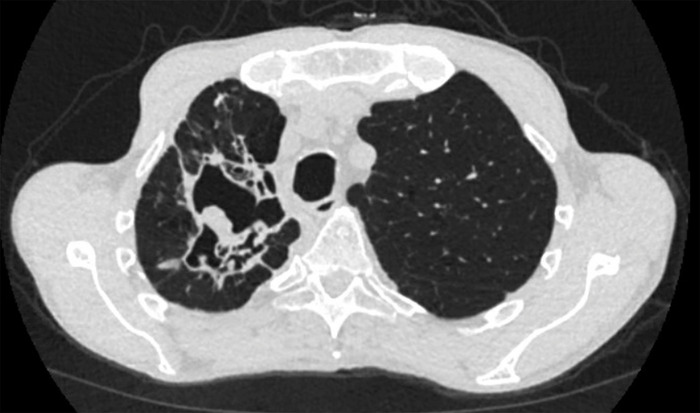
Follow-up chest computed tomography after 5 weeks, transverse view of lower lobes, shows near complete clearing of bibasilar opacities. Bilateral partially calcified nodules and amorphous/nodular opacities (more on the right than on the left) are visible, as well as bilateral bronchiectasis (also more on the right than on the left).

**Figure 7.**
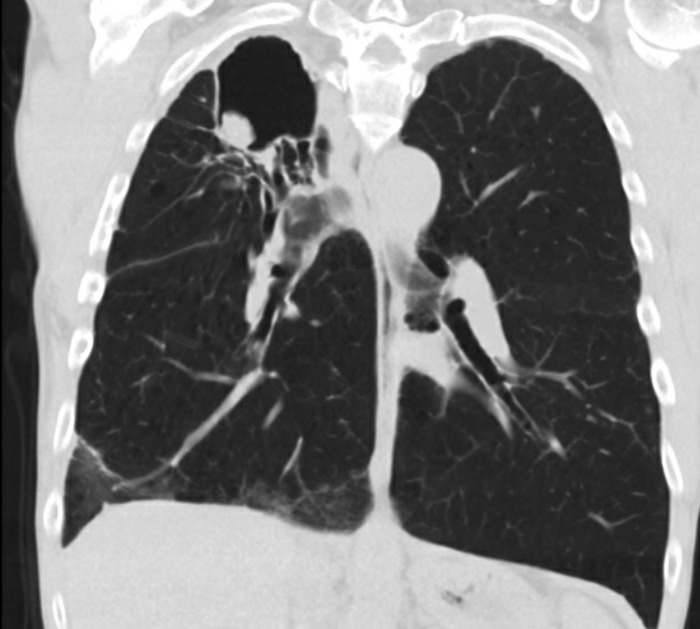
Follow-up chest computed tomography after 5 weeks, coronal view, shows stable borderline mediastinal lymphadenopathy.

Eventually, the patient was able to tolerate breathing room air. On day 4, he was deemed stable and discharged home. He was instructed to take a prednisone taper of 40 mg daily for 7 days, 30 mg daily for 7 days, and 20 mg daily for 7 days. The patient was counseled about the detriments of smoking, his current occupation, and wearing proper equipment. However, he only intermittently used the face mask at work, presented again in 2 weeks with similar symptoms, and responded well to steroids. A 5-week follow-up chest CT showed resolution of the bibasilar opacities, and the right upper lobe intracavitary lesion was stable (Figures 5, 6, and 7).

## DISCUSSION

HP, also known as extrinsic allergic alveolitis, is a pulmonary disease with large knowledge gaps, including a complete list of inciting antigens, the specific immune response behind producing HP, a method for definitive diagnosis, and treatment options to increase long-term survival.^1^ HP can be classified as acute, subacute, and chronic. Acute HP develops through intense, intermittent or recent antigen exposure with a symptom onset of a few hours.^1,2^ Subacute HP occurs after weeks or months of continual exposure. The chronic form may be insidious and only present after a trigger arises, such as pneumonia or influenza.^3^ Our patient's illness was attributed to subacute HP, as he presented with symptoms of 1-week duration after exposure to the trigger for 6 weeks. Moreover, he experienced rapid symptomatic resolution with minimal medical intervention.

Metalworking fluids cool metals, preventing corrosion and damage to machines, and are especially vital in industries involved in metal shaping.^5^ Metalworking fluids with isocyanate chemicals are strongly linked as HP antigens that cause chemical worker's lungs, a specific type of HP.^1,6^ Metalworking fluids are made of many components, including water, mineral oil, emulsifiers, extreme-pressure additives, and corrosion inhibitors.^7^ Fire-retardant spray, classified as a nonmetalworking fluid spray, contains a mixture of water, detergent, and foaming chemicals. These foaming chemicals are made with an ammonium-based fertilizer, a thickener, and a corrosion inhibitor.^8^ Other nonmetalworking fluid sprays have been associated with HP (Table 2).^9-13^ We propose that the corrosion inhibitors might be the causative agent in HP because they are a common ingredient in both metalworking fluids and nonmetalworking fluid sprays. To our knowledge, our case is the first report of a fire-retardant spray causing HP. Thus, we propose that causes of chemical worker's lungs should include exposures to both metalworking fluids and nonmetalworking fluid sprays.

**Table 2.** Nonmetalworking Fluid Sprays Causing Hypersensitivity Pneumonitis

Study	Age, years	Exposure	Treatment
Akimoto et al, 1992^9^	68	Paint spray	Long-term steroids
Bando et al, 1993^10^	50	Spray paint	Steroids
Hashizume et al, 2001^11^	47	Automobile spray paint	Steroids
Charles et al, 1976^12^	50	Polyurethane foam spray	Steroids and contact avoidance
	50	Polyurethane foam spray	Contact avoidance
	61	Polyurethane foam spray	Contact avoidance
	46	Polyurethane paint	Contact avoidance
Stringer et al, 1977^13^	33	Aerosol hairspray	Contact avoidance

The progression rate of HP, which is the development of HP after exposure to a known inciting antigen, is reported to be only 5% to 15%.^1^ Antigen characteristics and individual susceptibility, including genetic and environmental factors, can greatly affect the progression rate of HP. One documented risk factor is previous nonspecific lung inflammation.^1^ Our patient had several chronic pulmonary inflammatory conditions, including emphysema and chronic *M malmoense*. The current proposed pathogenesis, which is not clearly defined, is that inhaled soluble antigens with diameters <3 μm enter the distal bronchial tree and alveoli. From there, the antigens are transported by the lymphatic system to the hilar nodes where they bind with immunoglobulin G (IgG) antibodies to produce immune complexes.^14,15^ Moreover, the hilar lymph nodes have been specifically associated with generating an adequate antibody-mediated immune response. These complexes activate the complement cascade, eventually producing the C5 factor that activates macrophages to secrete chemokines and cytokines. Thereafter, neutrophils, T lymphocytes, and monocytes are attracted to the site of inflammation.^1,14,15^

Nicotine from cigarettes has been reported to decrease IgG and cytokine levels, so it can be seen as protective against HP. However, if HP develops in a smoker as it did in our patient, it is usually chronic and characterized by recurrent acute exacerbations with a higher mortality compared to nonsmoking patients.^1,3^ We theorize that the higher mortality in smoking patients is because the decreased IgG level results in symptoms not manifesting until the disease has reached an irreversible point. In contrast to the typical IgG mechanism, we noted a significantly elevated IgE level at 2,393 IU/mL in our patient. Because our patient did not have a history of asthma, atopic dermatitis, or allergies, we considered this laboratory value to be acute. Although rare, IgE has been linked with HP. A flooring department employee developed HP and had an IgE level of 1,997 IU/mL.^4^ In another case, a patient whose job required spending time in a walk-in refrigerator had an IgE level of 440 IU/mL.^16^ HP attributed to an IgE immune response has been described as a severe type; however, the pathogenesis behind the correlation between the two is not well understood.^4^ We postulate that this correlation could be attributed to the specific granules within eosinophils vs a combination of IgG and IgE responses.

Patients with HP typically present with acute infection-like symptoms,^15^ so a high index of clinical suspicion is necessary to consider HP. No established set of diagnostic criteria for HP exists, but pathways have been proposed. D'souza and Donato suggest conducting a thorough history to identify potential exposures and a physical examination to evaluate for coarse inspiratory rales or inspiratory wheezes.^16^ High-resolution CT of the chest would show a pattern of centrilobular diffuse micronodular, ground-glass opacification and mosaic attenuation in the upper and middle lobes. Fibrosis may present itself as reticulation, architectural distortion, and traction bronchiectasis with or without honeycombing appearance.^17^ An HP panel uses serum precipitins to measure the antibody response to specific pathogens.^18^ Positive antibodies can be present in asymptomatic patients, so clinically correlating the results is important.^19^ As shown in previous studies, an HP panel is useful in suggesting a diagnosis, but diagnoses cannot be excluded or included by the panel itself.^1,14^ Thereafter, serum-specific IgGs can be drawn, and if positive with an elevated titer, the diagnosis of HP is likely.^17^ A specific inhalation challenge may be performed during which the patient is exposed to the antigen, and the clinician monitors for a response.^17^ Bronchoalveolar lavage via bronchoscopy can be analyzed for alveolar lymphocytosis, CD4/CD8 ratio, and pathogen-specific antibodies.^1,3,15,20^ As a last resort, a lung biopsy with histopathology report can show noncaseating granulomas, neutrophil and eosinophil infiltration of alveolar spaces, small vessel vasculitis, and diffuse inflammation.^19^ However, none of these features can yield a definitive diagnosis because of their lack of high specificity and sensitivity.^1,3,18^ In our case, the patient's social and medical history, physical examination, and laboratory findings led to the final diagnosis. Despite his positive HP panel, our patient was not diagnosed with bacterial or fungal HP, as the clinical picture did not fit the positive antibodies. The patient was offered the current options for definitive diagnosis, but he declined because he had symptomatic relief.

The mainstay of treatment for HP is contact withdrawal and avoidance of further antigen exposure, as symptoms can resolve rather quickly. Oral or systemic steroids can be used to expedite the recovery process for symptomatic relief but have no known long-term benefits.^1,3^ For oral prednisolone, the dosage in the literature varies (50 mg or 20 mg daily) as does duration (2 or 4 weeks).^3,15^ Chronic HP can lead to irreversible lung fibrosis, but as of 2021, no medications are available to decrease or delay this progression.^3^ Therefore, further studies are needed to determine the guideline-based treatment in these patients in terms of which patients need medications and what medications decrease mortality long term.

## CONCLUSION

This case illustrates that HP can develop via chemical fire-retardant spray, and patients with smoking history and elevated IgE should be evaluated for severe forms of the disease. Randomized controlled trials are needed to fill in the gaps of knowledge about HP.

## Notes

*This article meets the Accreditation Council for Graduate Medical Education and the American Board of Medical Specialties Maintenance of Certification competencies for Patient Care and Medical Knowledge.*
